# Plasticity of vertebral wedge deformities in skeletally immature patients with adolescent idiopathic scoliosis after posterior corrective surgery

**DOI:** 10.1186/s12891-016-1287-1

**Published:** 2016-10-12

**Authors:** Takahiro Makino, Takashi Kaito, Yusuke Sakai, Shota Takenaka, Kazuomi Sugamoto, Hideki Yoshikawa

**Affiliations:** 1 Department of Orthopaedic Surgery, Osaka University Graduate School of Medicine, 2-2, Yamadaoka, Suita, Osaka 565-0871 Japan; 2 Department of Orthopedic Biomaterial Science, Osaka University Graduate School of Medicine, 2-2, Yamadaoka, Suita, Osaka 565-0871 Japan

**Keywords:** Adolescent idiopathic scoliosis, Wedging, Vertebral body, Reshaping

## Abstract

**Background:**

Vertebral bodies in patients with adolescent idiopathic scoliosis (AIS) usually have frontal wedge deformities. However, the plasticity of the deformed vertebrae in skeletally immature patients is unknown. The purpose of our study was to clarify the plasticity of vertebral deformities in skeletally immature patients with AIS by using in vivo three-dimensional (3D) analysis.

**Methods:**

Ten female patients with AIS (mean age, 12.2 years; three patients, Lenke type 1; five patients, type 2; two patients, type 5) who underwent posterior fusion and whose Risser grade was ≤3 at surgery were included. Using computed tomography images (0.625-mm slice thickness) obtained 1 week and 1 year postoperatively, a total of seventy-three 3D bone models of vertebrae was made. The 3D bone models were made between the upper and lower end vertebrae within the main thoracic curve for patients with Lenke types 1 and 2 scoliosis, whereas they were made within the thoracolumbar/lumbar curve in patients with Lenke type 5 scoliosis. The height of the concave and convex sides in the anterior, middle and posterior parts of the vertebral bodies was measured using the original digital viewer, and the vertebral height ratio (VHR: concave/convex) was calculated. VHRs at 1 week and 1 year postoperatively were compared using the Wilcoxson signed-rank test. Differences were considered statistically significant at *p* < 0.05.

**Results:**

VHR of the end vertebrae (*n* = 20) did not change postoperatively for any parts of the vertebral bodies. VHR of the vertebrae in the apical region (*n* = 28) also remained unchanged postoperatively. In contrast, VHR of the other vertebrae (*n* = 25) increased significantly in the anterior part postoperatively (from 0.938 to 0.961, *p* = 0.006).

**Conclusions:**

The wedge deformity of vertebral bodies showed a reshaping potential towards a symmetrical configuration in the region other than end and apex, although no plasticity of the vertebrae was observed in the apical region even in skeletally immature patients with AIS.

## Background

Adolescent idiopathic scoliosis (AIS) is defined as scoliosis with an onset between ages 10 and 18 years [1, 2]. Although AIS is a relatively common spinal disorder affecting 1–3 % of children, its etiology remains unknown [1, 2]. Because the onset and progression of AIS is at or around puberty when the osseous growth of vertebrae occurs in spurts, the unbalanced axial load caused by primary scoliotic changes can affect the growth and morphology of adjacent vertebrae.

Recent morphological analysis of the vertebrae in patients with AIS revealed that the vertebral bodies have three-dimensional (3D) deformities (e.g. wedging and torsion) and that these deformities are primarily oriented in the frontal plane [3–6]. It is still controversial whether these deformities are secondary change caused by asymmetrical vertebral loading or primary changes caused by aberrant asymmetrical vertebral growth [7]. If deformities are secondary, reshaping of the vertebral bodies can occur postoperatively, which will remove asymmetrical loading. However, little is known about the plasticity of vertebral deformities in AIS.

Posterior corrective surgery in AIS attenuates the excessive axial loading in the concave side by restoring physiological vertebral alignment. We hypothesized that the secondary deformity caused by asymmetrical axial loading can be reshaped postoperatively in skeletally immature AIS patients. Therefore, the purpose of our study was to clarify the plasticity of vertebral wedge deformities in skeletally immature AIS patients using in vivo 3D analysis.

## Methods

Our study was a retrospective review of a radiological database of patients with AIS who underwent corrective surgery, and was approved by the Research Ethics Committee of Osaka University Hospital (no. 15098).

**Fig. 1:**
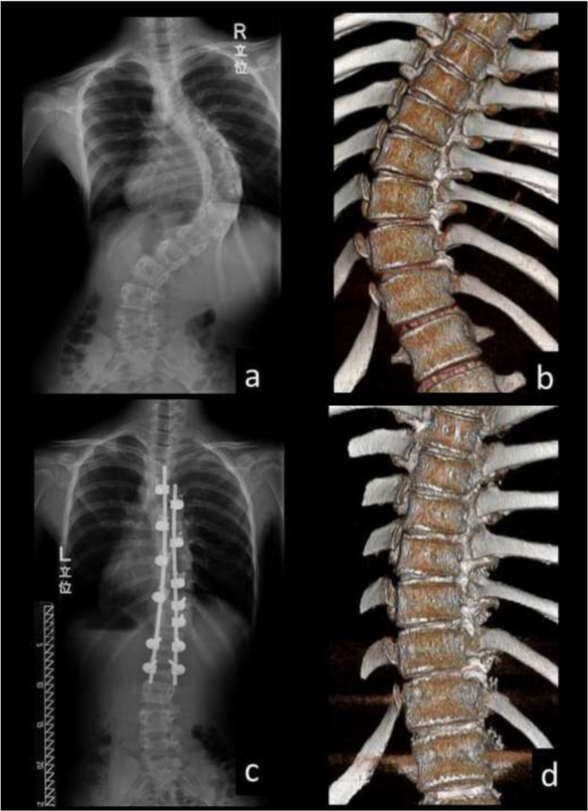
Preoperative (**a**, **b**) and postoperative (**c**, **d**: 1 week postoperatively) full-length standing posteroanterior radiographs and 3D reconstructed CT images in the main thoracic curve of a 12-year-old female patient (Patient No. 5 in Table 1)

**Fig. 2:**
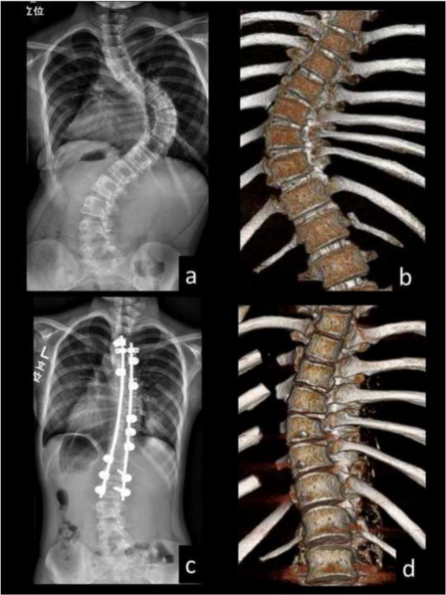
Preoperative (**a**, **b**) and postoperative (**c**, **d**: 1 week postoperatively) full-length standing posteroanterior radiographs and 3D reconstructed CT images in the main thoracic curve of an 11-year-old female patient (Patient No. 6 in Table 1). The 3D-CT images demonstrated that the opening of disc spaces in the apical region was observed at the concave side

We included 10 female skeletally immature patients (Risser grade ≤3) who underwent posterior corrective spinal fusion surgery for AIS between 2008 and 2012. The mean age at the time of the surgery was 12.2 years (range, 10–14 years). In brief, the surgical procedures were as follows. Only by posterior approach, instrumentation and correction of curve were performed. Unilateral pedicle hook placement at upper and middle thoracic spine and bilateral or unilateral pedicle screw insertion at lower thoracic and lumbar spine were performed. All the inferior articular processes within fusion segments were resected for posterior release. The correction of curve was performed by a rod rotation maneuver and distraction at the concave side, without direct vertebral rotation. All surgeries were performed by a single surgery team of our spine care unit. COLORADO 2^TM^ Spinal System (Medtronic Sofamor Danek Inc., Memphis, TN) was used for all surgeries (Figs. 1 and 2).

### Radiographic assessments

The type of scoliosis of each patient was classified according to Lenke classification on the basis of preoperative full-length standing posteroanterior and lateral radiographs [8]. If an apex was located at an inter-vertebral disc, the adjacent upper and lower vertebral bodies were defined as apical vertebrae. Cobb angles of the main thoracic (MT) and thoracolumbar/lumbar (TL/L) curves and Risser grades were digitally measured on a flat-panel monitor at our hospital using a built-in imaging software (Centricity WebDX; GE Healthcare Japan, Tokyo, Japan).

### Computed tomography (CT) assessments

The patients underwent CT scans within 3 months preoperatively as well as 1 week and 1 year postoperatively. Aquilion ONE^TM^ (Toshiba Medical Systems Corporation, Tochigi, Japan) was used for the CT scans. The settings used for CT scans were a slice thickness of 0.5 mm, a tube voltage of 120 kV and a tube current of 60 mA. The mean dose length product per single CT scan was 365.66 ± 192.22 mGy · cm.

#### Segmentation and creation of a 3D bone surface model

The obtained image data of the CT scans, in Digital Imaging and Communications in Medicine standard format, were imported into a 3D image processing workstation (Synapse Vincent; Fujifilm Holdings Corporation, Tokyo, Japan). The segmentation and creation of a 3D bone surface model of each vertebra were performed semi-automatically. The contour of each vertebra was extracted at a threshold of 2000 Hounsfield units for window width and 150 Hounsfield units for window level, as has been previously described [9, 10]. The 3D models were made between the upper and lower end vertebrae within the MT curve for patients with Lenke types 1 and 2 scoliosis, whereas they were made within the TL/L curve in patients with Lenke type 5 scoliosis. As a result, 73 3D models of vertebrae was made. Thereafter, the vertebral bodies were extracted semi-automatically from the 3D models of the vertebrae by removing posterior elements at the transitions between the vertebral bodies and pedicles.

#### Measurement of vertebral height and vertebral angle

**Fig. 3:**
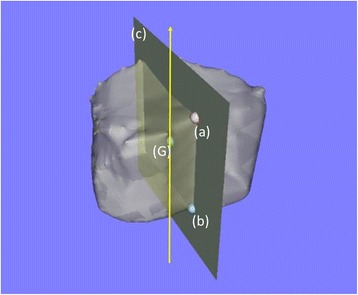
An example of the vertical axis and sagittal plane of a vertebral body. The vertical axis (*arrow*) was defined as the line containing the center of gravity (G) and parallel to the line connecting anterior edges of the vertebral foramen on the upper and lower vertebral end plates (a, b). The sagittal plane (c) contained the center of gravity and anterior edges of the vertebral foramen on the upper and lower end plates

**Fig. 4:**
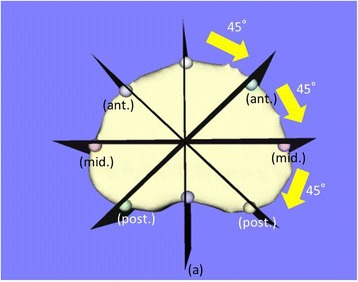
The intersection points of the rim of the lower endplate of a vertebral body and the rotated sagittal plane. A sagittal plane (a) was rotated on the vertical axis at 45° intervals. The anterior two intersection points (ant.) were used for assessing the anterior part of the vertebral body, the middle two intersection points (mid.) for the middle part and the posterior two intersection points (post.) for the posterior part

**Fig. 5:**
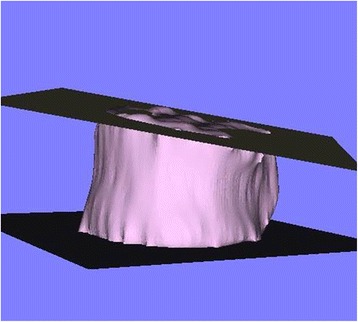
An example of the approximated planes of the upper and lower endplates of a vertebral body. These planes were calculated automatically by custom-made software (Orthopedic Viewer, Osaka University)

The following processing and measurements were performed semi-automatically using the original digital viewer (Orthopedic Viewer; Osaka University). The center of gravity of each vertebral body was calculated, and the vertical axis of each vertebral body was defined as the line containing the center of gravity that runs parallel to the line connecting the anterior edges of the vertebral foramen on the upper and lower vertebral end plates (Fig. 3). Sagittal planes of each vertebral body, which contained the center of gravity and anterior edges of the vertebral foramen on the upper and lower end plates, were made (Fig. 3), and were then rotated on the vertical axis at 45° intervals (Fig. 4). The intersection points of the rim of the lower endplate of each vertebral body and the rotated sagittal planes were then plotted (Fig. 4). The anterior two intersection points were used for assessing the anterior part of the vertebral body, the middle two intersection points for assessing the middle part and the posterior two intersection points for the posterior part (Fig. 4). Moreover, approximated planes of the upper and lower endplates of each vertebral body were made (Fig. 5). We measured and calculated the following parameters.

**Fig. 6:**
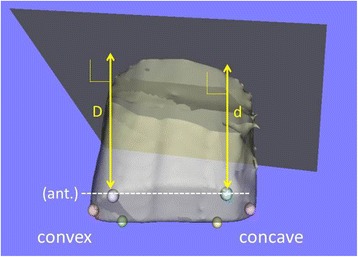
An example of vertebral height (VH). VH was defined as the distance between each intersection point and the approximated plane of the upper vertebral end plate (D, d). VHR was defined as the ratio of the VH of the concave side to that of the convex side (e.g. VHR at anterior = d/D)
- Disc opening angle (DOA)
  As the index of the opening change of the discs in the coronal plane for each vertebral body at the concave side, DOA was measured as follows. First, the coronal plane of each vertebral body was made by rotating the sagittal plane of each vertebral body by 90° around the vertical axis. Then, in the coronal plane of the lower vertebral body adjacent to the disc, the angle between the approximated planes of the lower endplate of the upper vertebral body and the upper endplate of the lower vertebral body adjacent to the disc (‘plus’ indicates that the opening is at the convex side) was measured as the disc angle (Fig. 7). DOA was calculated using the following formula:

- Vertebral height (VH) and Vertebral height ratio (VHR)
  VH was defined as the distance between each intersection point and the approximated plane of the upper vertebral end plate (Fig. 6). VHR, defined as the ratio of the VH of the concave side to that of the convex side, was used as the index of wedge deformity of the vertebral bodies in the coronal plane. VHR was assessed at the anterior, middle and posterior parts of each vertebral body, respectively. A value of VHR getting closer to 1 implies that the upper and lower endplates of the vertebral bodies in the coronal plane are closer to being parallel.

**Fig. 7:**
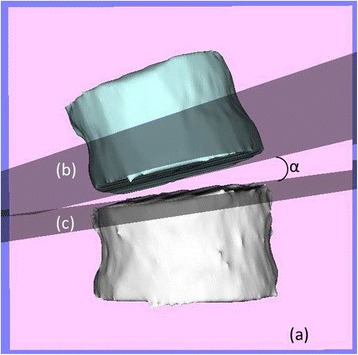
An example of coronal disc angle (CDA). CDA (α) was defined as the angle in the coronal plane of the lower vertebral body adjacent to the disc (a), between the approximated planes of the lower endplate of the upper vertebral body (b) and the upper endplate of the lower vertebral body (c) adjacent to the disc

$$ \begin{array}{l}\mathrm{D}\mathrm{O}\mathrm{A}\kern0.5em =\kern0.5em \left[\right(\mathrm{preoperative}\ \mathrm{disc}\ \mathrm{angle}\ \mathrm{of}\ \mathrm{the}\ \mathrm{adjacent}\ \mathrm{upper}\ \mathrm{disc}\kern0.5em -\kern0.5em \mathrm{postoperative}\ \left(1\mathrm{w}\right)\ \mathrm{disc}\ \mathrm{angle}\ \mathrm{of}\\ {}\mathrm{the}\ \mathrm{adjacent}\ \mathrm{upper}\ \mathrm{disc}\left)\kern0.5em +\kern0.5em \right(\mathrm{preoperative}\ \mathrm{disc}\ \mathrm{angle}\ \mathrm{of}\ \mathrm{the}\ \mathrm{adjacent}\ \mathrm{lower}\ \mathrm{disc}\kern0.5em -\kern0.5em \mathrm{postoperative}\\ {}\left(1\mathrm{w}\right)\ \mathrm{disc}\ \mathrm{angle}\ \mathrm{of}\ \mathrm{the}\ \mathrm{adjacent}\ \mathrm{upper}\ \mathrm{disc}\left)\right]/2\end{array} $$

A higher DOA means that the discs adjacent to the vertebral body largely open at the concave side postoperatively.

### Statistical analysis

The postoperative changes of VHR and DOA were analyzed statistically in end vertebrae (EV), vertebrae in the apical region (AV: an apical vertebra and adjacent upper and lower vertebrae. If an apex was located at an inter-vertebral disc, the upper and lower vertebrae adjacent to the disc were defined as AV), and the other vertebrae (OV: vertebrae other than EV and AV), respectively. Statistical analysis was performed using IBM SPSS Statistics Version 22 (IBM, Armonk, NY, USA). The Wilcoxson signed-rank test was used to compare VHR between 1 week and 1 year postoperatively. The Scheffe test was used to compare DOA among the EV, AV and OV. Intra-observer and inter-observer agreement for measurements of VH and disc angle were assessed with intra-class correlation coefficients based on the blinded evaluation of these parameters in two patients (VH, 120 points; disc angle, 18 discs). For the analysis of intra-observer reliability, the first author (T.M.) evaluated these parameters twice with a 2-week interval. For the analysis of inter-observer reliability, two spinal surgeons (T.M. with a 6-year experience of CT analysis of scoliosis, and Y.S. with a 3-year experience) evaluated these parameters. Differences were considered statistically significant at *p* < 0.05.

## Results

Based on Lenke classification of AIS, three patients were type 1, five patients were type 2 and two patients were type 5. The mean Cobb angle of the MT curve in patients with type 1 and type 2 was 66.3° ± 19.4° (range, 39°–97°), and that of the TL/L curve in patients with type 5 was 56.5° ± 6.5° (range, 50°–63°). The mean Risser grade was 1.8 ± 1.2 (range, 0–3). The demographic data of each patient are shown in Table 1.

**Table 1:** Patients’ demographic data

				Characteristics of main structural curve [Main thoracic curve (Patients 1–8), Thoracolumbar/lumbar curve (Patients 9 and 10)]			
Patient no.	Lenke type	Risser grade	Fusion area	End	Apex	Cobb’s angle (preoperative; °)	Cobb’s angle (postoperative; °)
1	1A-	3	T4–T11	T5/T11	T8/T9	44	37
2	1B-	2	T5–T12	T5/T11	T8	39	28
3	1AN	3	T4–T12	T5/T12	T9	61	26
4	2AN	3	T2–L2	T5/T12	T9	56	35
5	2AN	1	T4–L2	T6/L1	T10	92	24
6	2A+	1	T3–L2	T6/L1	T9	97	27
7	2CN	2	T3–L3	T6/T11	T8/T9	71	30
8	2CN	0	T2–L3	T5/T12	T9	70	28
9	5CN	3	T7–L3	T9/L3	T12	63	16
10	5CN	0	T10–L3	T10/L3	T12	50	9
Means ± SDs		1.8 ± 1.2				64.3 ± 18.0	26.0 ± 7.9

*SD* indicates standard deviation

**Fig. 8:**
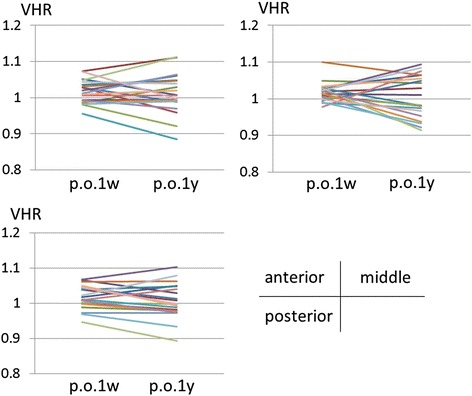
The changes of the vertebral height ratio (VHR) of the end vertebrae (*n* = 20) between 1 week and 1 year postoperatively. VHR of the end vertebrae did not change in any part

**Fig. 9:**
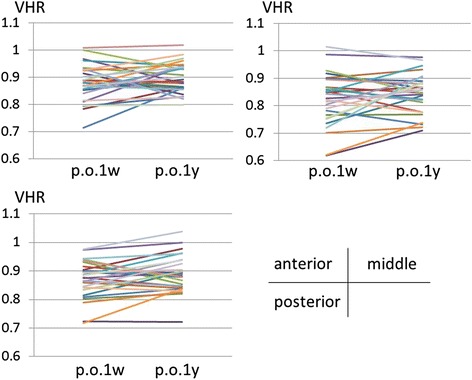
The changes of the vertebral height ratio (VHR) of the vertebrae in the apical region (*n* = 28) between 1 week and 1 year postoperatively. VHR of the vertebrae in the apical region did not change in any part

**Fig. 10:**
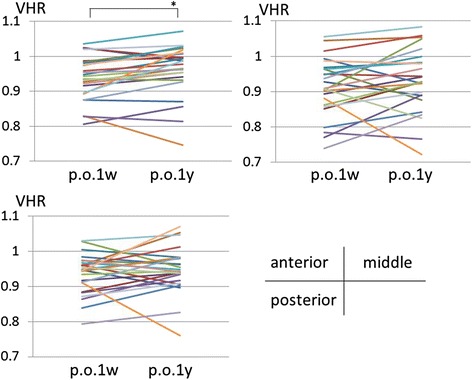
The changes of the vertebral height ratio (VHR) of the vertebrae other than the end vertebrae and vertebrae in the apical region (*n* = 25) between 1 week and 1 year postoperatively. VHR of the vertebrae other than the end vertebrae and vertebrae in the apical region at the middle and posterior part did not change postoperatively; however, VHR at the anterior part increased. (*p = 0.006)

VHR of the EV (*n* = 20) did not change between 1 week and 1 year postoperatively for the anterior, middle and posterior parts of the vertebral bodies (Table 2, Fig. 8). VHR of the AV (*n* = 28), where the most severe wedge deformities were observed, did not change during the observation period (Table 2, Fig. 9). However, VHR in the OV (*n* = 25) significantly increased (i.e. remodeled towards a symmetrical configuration) between 1 week and 1 year postoperatively at the anterior part (from 0.938 to 0.961, *p* = 0.006, [1 - β] = 0.758) (Table 2, Fig. 10).

**Table 2:** Measurements of vertebral height ratio (VHR)

	1 week postoperatively	1 year postoperatively	*p* value
EV (*n* = 20)			
Anterior	1.017 ± 0.031	1.012 ± 0.055	0.823
Middle	1.018 ± 0.026	1.006 ± 0.057	0.314
Posterior	1.018 ± 0.033	1.007 ± 0.048	0.126
AV (*n* = 28)			
Anterior	0.881 ± 0.066	0.898 ± 0.054	0.179
Middle	0.823 ± 0.093	0.845 ± 0.071	0.246
Posterior	0.871 ± 0.064	0.889 ± 0.064	0.111
OV (*n* = 25)			
Anterior	0.938 ± 0.061	0.961 ± 0.071	0.006
Middle	0.912 ± 0.081	0.932 ± 0.089	0.088
Posterior	0.936 ± 0.055	0.948 ± 0.065	0.183

Values are expressed as means ± standard deviations

Wilcoxson signed-rank test

*EV* indicates end vertebrae, *AV* vertebrae in the apical region, *OV* vertebrae other than EV and AV

DOA was -0.12° ± 2.70° at the EV, 6.01° ± 3.33° at the AV and 3.38° ± 2.30° at the OV. DOA at the AV was greater than that at the EV and OV (vs. EV, *p* < 0.001; vs. OV, *p* = 0.007). DOA at OV was also greater than that at the EV (*p* < 0.001).

Intra-class correlation coefficients for intra-observer and inter-observer reliabilities were 0.996 (95 % confidence interval, 0.994–0.997) and 0.990 (0.986–0.993) for VH, and 0.981 (0.951–0.993) and 0.960 (0.899–0.985) for disc angle, respectively.

## Discussion

In accordance with our hypothesis, our study revealed that vertebrae with wedge deformities in skeletally immature AIS patients have remodeling potential towards a symmetrical configuration at the anterior part of the OV. However, remodeling was not observed at the AV where the wedge deformities were most severe, although distraction of the adjacent disc spaces at the concave sides was confirmed by the increased DOA.

It is well known that mechanical loading influences the longitudinal growth of the long bones and vertebrae. Growth is retarded by increased mechanical compression and accelerated by decreased loading, and this phenomenon is explained as the Hueter–Volkmann Law [11]. Using a rat-tail model, Stokes et al. [12] showed that the longitudinal growth of vertebrae was suppressed under compression force and accelerated under distraction force. In patients with scoliosis, asymmetrical loading in the inter-vertebral disc has been proved in vivo, and loading in the concave annulus has been shown to be greater than in the convex annulus [13, 14]. Therefore, in patients with scoliosis, this can cause a vicious cycle of asymmetrical loading and vertebral wedge deformities [7, 12]. Many researchers have shown that vertebral wedge deformities exist in patients with scoliosis [3–6, 15]. Recent 3D morphometric studies revealed that the severity of wedge deformities correlated with the Cobb angle and that this wedge deformity was greater in the apical region [4, 15].

It is unclear as to why reshaping of the wedged vertebrae did not occur in the apical region, even though more correction force was applied and the correction of the coronal curve as measured by DOA was not less than that in the other regions. One hypothesis is that the epiphyseal growth plate of a vertebral body can be injured at the concave side of the vertebrae at the apical lesion by excessive loading. In a morphometric study regarding vertebral wedge deformities in AIS, Modi et al. [15] suggested that the load distribution was concentrated maximally at the apex. A histomorphological study of spinal growth plates in AIS by Wang et al. [16] showed that no signs of growth activity were observed in the growth plates at the concave side of the apical vertebrae compared to the convex side of the apical vertebrae and the concave side of the upper or lower end vertebrae. They also showed that the proliferation and apoptosis of chondrocytes in the proliferative and hypertrophic zones were inhibited at the concave side of the apical vertebrae compared to the convex side of the apical vertebrae and the concave side of both the upper and lower end vertebrae. This supports our finding that reshaping did not occur at the apical wedged vertebrae.

Another hypothesis is that the loss of plasticity at the apical vertebrae in patients with AIS is caused by primary factors. *GPR126* and *BNC2* were reportedly two of the candidate susceptibility genes for AIS, and *GPR126* knockdown or *BNC2* overexpression in zebrafish has been shown to cause delayed ossification of the developing spine and scoliosis [17, 18]. Growth arrest at the epiphyseal growth plates at the concave side of the apical vertebrae in patients with AIS could be induced by the asymmetrical expression of some genes regulating spine ossification or some disorders such as osteochondrosis. Vertebral wedge deformities have been observed even in the early stages of mild scoliosis [5, 6]. This also supports the fact that primary factors can affect vertebral body growth in AIS.

Vertebral rotation is one of the concerns in vertebral morphometric studies on AIS. It is well known that the vertebrae in patients with AIS rotate in the axial plane and that the rotation increases gradually from neutral vertebrae to apical vertebrae [19, 20]. Recent studies have revealed that an increase in vertebral rotation in the thoracic spine could induce the loss of local kyphosis in the ‘true’ 3D sagittal plane [21, 22]. Using 3D analysis, Hattori et al. [23] showed that local lordosis could occur at the thoracic apical lesion. In our study, we defined the 3D sagittal plane of each vertebral body in order to consider vertebral rotation. Our study showed that VHR of the OV increased at the anterior part; however, VHR at the middle and posterior parts remained unchanged. This could be attributed to the posterior spinal fusion procedure. However, as described above, local lordosis or loss of local kyphosis can occur even in the thoracic spine in AIS, and these abnormal sagittal alignments could result in epiphyseal growth plate injury at the middle and posterior parts of the vertebral bodies according to scoliosis progression.

Though the post hoc analysis revealed that the statistical power (1 – β) in the analysis of the change of VHR in the OV at the anterior part was relatively high, the primary limitation of our study is the small sample size. Therefore, we were unable to show differences in the plasticity of the vertebral bodies between the thoracic spine and the lumbar spine. It is because skeletally immature patients with AIS often undergo anterior corrective fusion surgery, and patients who undergo only posterior corrective surgery are relatively rare. Moreover, the used implant and correction maneuver for scoliosis have been changed since 2013 in our institution. Because these changes could affect the morphology of vertebral bodies, the patients who underwent surgery after 2013 were excluded. Further prospective studies are needed to clarify this issue.

## Conclusions

The wedge deformity of vertebral bodies in the coronal plane could be reshaped at the region other than the end and apex after posterior corrective surgery. In contrast, the vertebrae in the apical region had no plasticity even though more distraction force was applied to the concave side than the other regions. With regard to the clinical relevance of our findings, the evaluation of vertebral wedge deformities may represent a possible measure of the effect of brace treatments because the correction of asymmetrical loading to the vertebrae under brace treatments can induce vertebral reshaping in skeletally immature patients.

## Abbreviations

3D: Three-dimensional
AIS: Adolescent idiopathic scoliosis
AV: An apical vertebra and adjacent upper and lower vertebrae
CT: Computed tomography
DOA: Disc opening angle
EV: End vertebrae
MT: Main thoracic
OV: Vertebrae other than EV and AV
TL/L: Thoracolumbar/lumbar
VH: Vertebral height
VHR: Vertebral height ratio
